# Phylogeny and biogeography of African Murinae based on mitochondrial and nuclear gene sequences, with a new tribal classification of the subfamily

**DOI:** 10.1186/1471-2148-8-199

**Published:** 2008-07-10

**Authors:** Emilie Lecompte, Ken Aplin, Christiane Denys, François Catzeflis, Marion Chades, Pascale Chevret

**Affiliations:** 1 UMR CNRS 5202, Origine, Structure et Evolution de la Biodiversité, Département Systématique et Evolution, Muséum National d'Histoire Naturelle, 55 rue Buffon, 75005 Paris, France; 2 UMR CNRS/UPS 5174 "Evolution et Diversité Biologique" EDB, Université Paul Sabatier, Bat. 4R3, 118 route de Narbonne, 31062 Toulouse cedex 9, France; 3 Australian National Wildlife Collection, CSIRO Division of Sustainable Ecosystems, GPO Box 284, Canberra, ACT 2601, Australia; 4 Laboratoire de Paléontologie, Phylogénie et Paléobiologie – CC064, Institut des Sciences de l'Evolution (UMR 5554/CNRS), Université Montpellier II, Place E. Bataillon, 34 095 Montpellier Cedex 05, France; 5 Equipe Zoologie Moléculaire, Institut de Génomique Fonctionnelle de Lyon, Université de Lyon, CNRS, INRA, ENS de Lyon 46, Allée d'Italie 69007 Lyon, France

## Abstract

**Background:**

Within the subfamily Murinae, African murines represent 25% of species biodiversity, making this group ideal for detailed studies of the patterns and timing of diversification of the African endemic fauna and its relationships with Asia. Here we report the results of phylogenetic analyses of the endemic African murines through a broad sampling of murine diversity from all their distribution area, based on the mitochondrial cytochrome b gene and the two nuclear gene fragments (IRBP exon 1 and GHR).

**Results:**

A combined analysis of one mitochondrial and two nuclear gene sequences consistently identified and robustly supported ten primary lineages within Murinae. We propose to formalize a new tribal arrangement within the Murinae that reflects this phylogeny. The diverse African murine assemblage includes members of five of the ten tribes and clearly derives from multiple faunal exchanges between Africa and Eurasia. Molecular dating analyses using a relaxed Bayesian molecular clock put the first colonization of Africa around 11 Mya, which is consistent with the fossil record. The main period of African murine diversification occurred later following disruption of the migration route between Africa and Asia about 7–9 Mya. A second period of interchange, dating to around 5–6.5 Mya, saw the arrival in Africa of *Mus *(leading to the speciose endemic *Nannomys*), and explains the appearance of several distinctive African lineages in the late Miocene and Pliocene fossil record of Eurasia.

**Conclusion:**

Our molecular survey of Murinae, which includes the most complete sampling so far of African taxa, indicates that there were at least four separate radiations within the African region, as well as several phases of dispersal between Asia and Africa during the last 12 My. We also reconstruct the phylogenetic structure of the Murinae, and propose a new classification at tribal level for this traditionally problematic group.

## Background

Rodents are the most speciose mammalian order and comprise almost half of all mammalian species diversity [1]. Within Rodentia, the most diverse assemblage is the superfamily Muroidea, with a global membership of 1300 living species and a natural distribution that includes all continents except Antarctica and all but the most remote islands. This remarkable group also includes the commensal rats and mice, long despised as human pests and agents of disease [2], but now highly valued as model organisms for research related to human health [3,4].

Not surprisingly, morphology-based classifications of muroid rodents were beset by problems of parallel evolution, with many common adaptations evolving independently on different landmasses. Molecular phylogenetic analyses are much less constrained by this problem and recent studies using slowly evolving nuclear genes have done much to clarify the membership and structure of Muroidea [5-7]. Recent classifications of this group recognize five or six family level lineages [7,8]. The speciose family Muridae Illiger, 1811 (150 genera and 730 species) is divided by Musser and Carleton [8] into five subfamilies, of which the Murinae Illiger, 1811 is the most diversified (126 genera, 561 species). Within the family Muridae, there is strong molecular support for three subfamilies (Deomyinae, Gerbillinae, Murinae) [subfamily Leimacomyinae of Musser and Carleton [8] has not yet been surveyed], and for a link between Deomyinae and Gerbillinae, with these as a sister clade to Murinae (this latter subfamily encompassing otomyines) [5-7].

The subfamily Murinae has a natural distribution that spans the Old World, including all of Africa and Eurasia, and extending to Australia, New Guinea and many islands of the western Pacific (we do not consider here the human-mediated distribution of a few commensal rodents of the genera *Mus *and *Rattus *in the Americas and throughout oceanic islands). More than 500 species are currently recognised [8], with centers of diversity and endemism in each of Tropical Africa, Southeast Asia, and the Australo-Papuan region [9,10]. Despite the obvious significance of this group for biogeographic studies, previous molecular studies have either had specific regional foci (e.g. Africa [11-13]; Philippines: [14]; Australia: [15,16] ; Eurasia: [17,18]) or employed immunological methods of uncertain reliability [10]. These studies have encouraged regionally-based classifications at tribal or subfamilial level, especially within the Australasian and Philippine regions where various higher level groupings are sometimes recognized (e.g. Anisomyini, Conilurini, Hydromyini, Phloeomyinae, Pseudomyinae, Rhynchomyinae). In Africa, Ducroz et al. [12] designated a tribe Arvicanthini for one well-supported monophyletic group. The most recent, global classification of Murinae [8] abandons the tribal level of classification in favour of a less formal arrangement of genera into divisions, following and improving a system already employed by Misonne [9]. Specifically, Musser and Carleton [8] (2005: pages 902 – 905) organize the 126 genera of the subfamily Murinae into 29 divisions, and consider the living taxa *Myotomys*, *Otomys*, and *Parotomys *as members of the subfamily Otomyinae.

Africa supports more than 25% of all living murine species including representatives of 32 endemic genera [8]. All African murines are endemic at species level and only two genera are shared between Africa and Eurasia. One of these is the genus *Mus*, which is widespread across Eurasia and is represented in Africa by an endemic subgenus, *Nannomys*, the African pigmy mice [19-21]. The second is the primarily African genus *Myomyscus *which has one species (*M. yemeni*) native to the Arabian Peninsula. A single origin for all African Murinae, except possibly *Dasymys*, was proposed by Watts and Baverstock [22] based on their analyses of albumin microcomplement fixation. In contrast, Chevret's [23] studies using the DNA/DNA hybridization method found a minimum of three ancient African lineages within Murinae, each associated with Eurasian taxa. Later studies using direct sequencing methods supported the notion of polyphyly for African Murinae, e.g. [12-14,16,24]. Jansa et al. [14] identified three distinct groups: the 'Arvicanthines' (*sensu *Ducroz et al. [12]), a '*Praomys *group' (*sensu *Lecompte et al. [25]) and the genus *Malacomys*. The 'otomyines', a dentally distinctive African lineage with three genera (*Myotomys*, *Otomys*,*Parotomys*), are variously associated in molecular studies with either the *Praomys *group [10] or the arvicanthines [6,11,12,16,24]. Ducroz et al. [12] suggested recognition of this group at tribal rank, as Otomyini. However, Musser and Carleton [8] follow more traditional practice by recognizing a distinct subfamily Otomyinae within Muridae.

Numerous questions thus remain unresolved concerning the pattern and timing of African Murinae diversification. In particular, the relationships of the various African lineages with Asian genera are enigmatic, and the timing of most cladogenic events remains poorly resolved or understood. The latter issue is critical to understanding the history of faunal interchange via the Arabian plate following the collision of Africa with Asia around 16 and 20 Million years ago (Mya) [26,27]. Notably, the murine palaeontological record attests to the presence of some shared genera in Africa and Asia during the late Miocene and the Pliocene [28-30], but whether this is due to multiple faunal exchanges between Asia and Africa, to the presence of ancient shared lineages followed by vicariance, or else to convergent evolution, remains a matter of conjecture.

To more adequately assess the pattern and timing of faunal exchanges between Africa and Asia, it is necessary to first establish a more complete phylogenetic framework including all of the key African and Eurasian lineages, and then to derive reliable estimates of divergence times. The main objectives of our study are: (1) to provide a robust and comprehensive phylogeny of the extant African murines and to infer their relationships with the Asian Murinae using mitochondrial and nuclear gene sequences, (2) to provide a new systematic framework that accurately reflects the phylogeny of Murinae; (3) to estimate times of origin and diversification for the African murines lineages; and (4) to place this phylogeny in an historical and geographical context to gain insight into the origin and maintenance of African murine diversity.

## Results

### Phylogenetics

**Figure 1:**
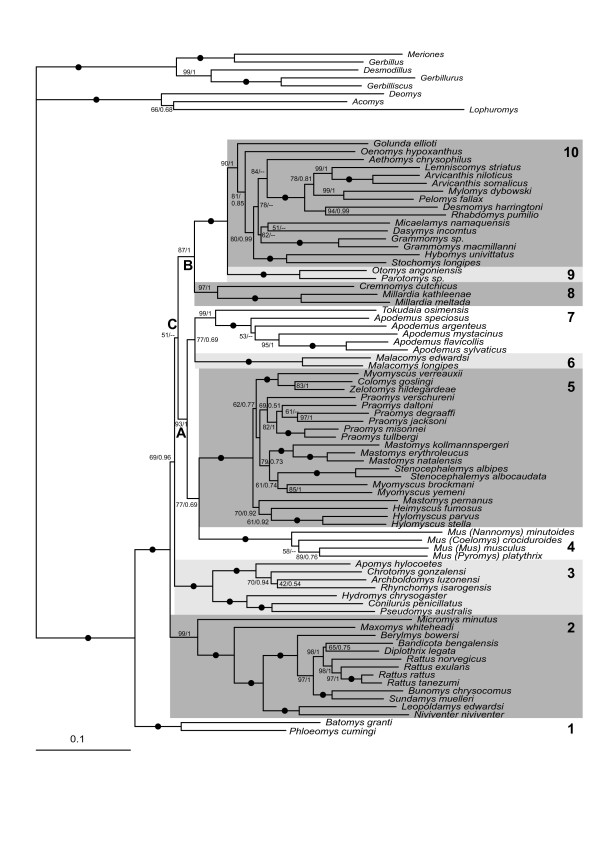
**Maximum likelihood tree for the combined dataset**. A black dot indicates that BP = 100 and PP = 1.0. Otherwise values are indicated as follow: BP/PP. An "-" indicates that MrBayes results support an alternative topology. The letters refer to the main groupings discussed in the text.

The final alignments included 1140 sites and 81 taxa for cyt *b*, 931 sites and 62 taxa for GHR, 1233 sites and 79 taxa for IRBP, and 3304 sites for 83 taxa for the concatenated dataset. The best-fitting substitution models were TVM+G+I for the GHR and IRBP data sets, and GTR+G+I for the cyt *b *and combined data set (Table 1). Analysis of the combined dataset produced a single ML tree (Figure 1, lnL = - 50270.78386), the supports obtained for each node and each gene are presented in the additional files 1 (ML analysis) and 2 (Bayesian analysis). Monophyly of Murinae is strongly supported but only with inclusion of the two 'otomyine' taxa (100% BP; 1.0 PP). Ten primary lineages can be recognized within Murinae, all with strong nodal support (Figure 1, BP ≥ 97%; PP = 1.0). African murines are polyphyletic and divided among five lineages. We here describe the different lineages to highlight the relationships among the African murines.

**Table 1:** Best model and estimated substitution parameter values.

Gene	Cytochrome b	IRBP	GHR	Combined data
Length (bp)	1140	1233	931	3304
Best model	GTR+G+I	TVM+G+I	TVM+G+I	GTR+G+I
πA	0.424	0.231	0.308	0.318
πC	0.324	0.278	0.252	0.287
πG	0.029	0.267	0.198	0.180
πT	0.223	0.224	0.242	0.215
rA-C	0.036	1.321	1.011	1.867
rA-G	3.821	6.415	5.880	4.316
rA-T	0.210	0.931	0.836	3.101
rC-G	0.251	0.621	1.376	0.632
rC-T	5.453	6.415	5.880	29.243
rG-T	1.000	1.000	1.000	1.000
α	0.475	0.918	0.640	0.290
Pinv	0.410	0.281	0.059	0.215

These values were estimated from maximum-likelihood analysis of each gene separately (cytochrome *b*, IRBP, and GHR, respectively) and of the combined data set.

The most basal lineage (Lineage 1) consists of the genera *Phloeomys *and *Batomys*, both Philippine endemics. There is very strong support (100% BP; 1.0 PP) for reciprocal monophyly of Lineage 1 and all other Murinae.

Among the remaining Murinae, the first lineage to diverge (Lineage 2, 99% BP; 1.0 PP) comprises one largely Southeast Asian clade, the *Rattus *group *sensu lato *of Verneau et al. [31], together with the Eurasian harvest mouse *Micromys*, again with strong support (99% BP; 1.0 PP). Within Lineage 2, *Micromys *is the first lineage to diverge, followed by *Maxomys*, then a sublineage consisting of *Niviventer *and *Leopoldamys *(100% BP; 1.0 PP), and finally, the *Rattus *group *sensu stricto *of Verneau et al. [31], comprising *Rattus, Berylmys, Bandicota, Diplothrix, Bunomys *and *Sundamys*. Almost all dichotomies within Lineage 2 are robustly supported (Figure 1).

The third lineage to diverge in the ML tree (Lineage 3, 100% BP; 1.0 PP) is a western Pacific group, divided into two well-supported sub-lineages: 1) a Philippine group (*Apomys*, *Archboldomys*, *Chrotomys*, and *Rhynchomys*: 100% BP; 1.0 PP); and 2) an Australo-Papuan group (*Hydromys*, *Conilurus *and *Pseudomys*: 100% BP; 1.0 PP). The relationships within Lineage 3 are mostly well resolved, save for some uncertainty over the branching order among *Apomys, Chrotomys *and *Rhynchomys*.

The fourth lineage consists of the genus *Mus *(Lineage 4, 100% BP; 1.0 PP), represented by all four subgenera including the African *Nannomys*. The relationships among the four *Mus *subgenera remain unresolved as the position of *Mus (Nannomys) minutoides *and *Mus *(*Coelomys*) *crociduroides *is unstable between ML and BI analyses [see additional files 1 and 2].

The fifth murine lineage is a diverse and robustly supported African assemblage (Lineage 5, 100% BP; 1.0 PP) that corresponds to the '*Praomys *group' of Lecompte et al. [13]. The monophyly of Lineage 5 is further supported by a shared insertion of 6 bp (TTGCCT) at position 893 of the GHR gene alignment. Although the basal nodes within Lineage 5 are poorly supported, it appears likely that *Mastomys *and *Myomyscus *are both paraphyletic. The order of branching between sublineages is unresolved and incongruent between ML and BI analyses [see additional files 1 and 2]. However, several terminal groups have strong support: 1) *Myomyscus verreauxii *+ *Colomys *+ *Zelotomys *(100% BP; 1.0 PP); 2) *Mastomys *(apart from *M. pernanus*) (100% BP; 1.0 PP); and 3) *Praomys *(apart from *P. verschureni*) (82% BP; 1.0 PP).

The sixth lineage (Lineage 6, 100% BP; 1.0 PP) consists of the genus *Malacomys*, the African swamp rats, here represented by two of the two recognized species.

The seventh murine lineage (Lineage 7, 99% BP; 1.0 PP) comprises the Eurasian genus *Apodemus *and the Ryukyu Island endemic genus *Tokudaia*.

The eighth lineage (Lineage 8, 97% BP; 1.0 PP) consists of the Indian genera *Cremnomys *and *Millardia*, the latter represented by two species.

The ninth murine lineage (Lineage 9, 100% BP; 1.0 PP) consists of the African 'otomyines' *Parotomys *and *Otomys*. As noted earlier, Musser and Carleton [8] included these taxa in a separate subfamily – Otomyinae.

The last murine lineage (Lineage 10, 90% BP; 1.0 PP) is very diverse and unites a large African assemblage of 'arvicanthines' (*sensu *Ducroz et al. [12]). Nodal support for 'arvicanthine' monophyly is moderately strong (90% BP; 1.0 PP). Branching order within this group is less well defined, with numerous distinct lineages apparent. The Indian bush rat genus *Golunda *occupies a basal position with moderate support (81% BP; 0.85 PP). Other near-basal lineages include *Oenomys*, *Stochomys *+ *Hybomys*, *Micaelamys*, *Grammomys, Aethomys, Dasymys *and a well supported (100% BP; 1.0 PP) sublineage which diversified later, consisting of *Arvicanthis, Lemniscomys, Mylomys, Desmomys, Rhabdomys *and *Pelomys*.

Relationships among the ten lineages are partially resolved under each of ML and BI but nodal support values are only moderate to strong. The best support is observed for a diverse Afro-Asian large group comprising Lineages 4 to 7, which we here call Clade A (93% BP; 1.0 PP). Monophyly of Clade A is further supported by an insertion of 6 bp (YGGAYG) at position 86 of the GHR alignment. Within this group, Lineages 6 and 7 are identified as sister lineages but with only moderate support (77% BP; 0.68 PP); and Lineages 4 and 5 form a second sister pair, also with only moderate support (77% BP; 0.69 PP). Lineages 8, 9 and 10, also representing a mix of both African and Asian taxa, are united on the ML tree with moderate to strong support (87% BP, 1.00 PP) in what is named Clade B. Lineages 9 and 10 are sister taxa, with a very strong nodal support (100% BP; 1.0 PP).

Clades A and B are identified as sister lineages on the ML tree, and build up what we refer to Clade C, albeit with very low support (51% BP). This clade C includes all the African murines. A different topology was obtained under BI [see additional file 2] in which Lineage 3 (Philippine and Australo-Papuan groups) forms the sister group of Clade B, once again with low support (0.60 PP). This was the only discrepancy in branching order among the primary lineages of Murinae observed between the two methods.

### Molecular divergence estimates

**Figure 2:**
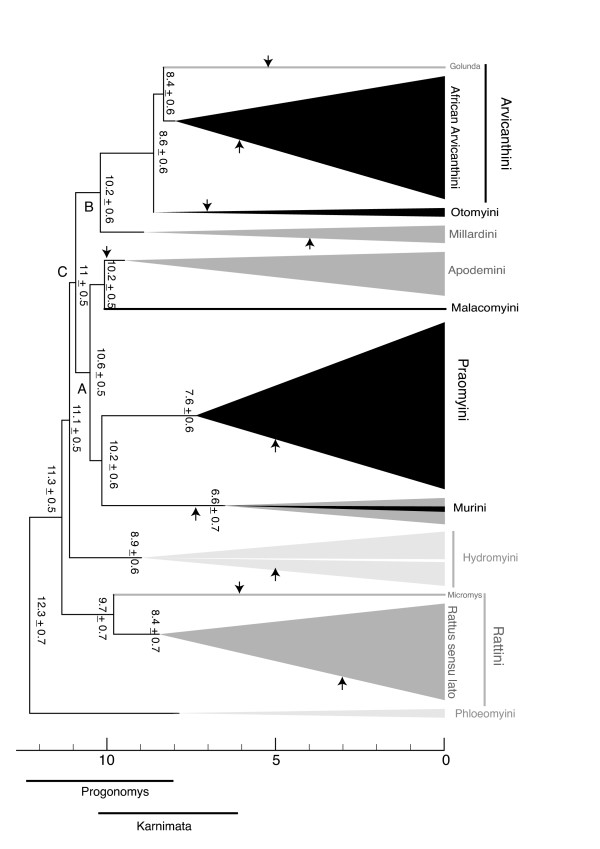
**Simplified chronogram with the main murine groups**. For each group the oldest fossil is indicated by an arrow according to [51,52,65,66,71,73,76,104,105,108,134-136]. Black area represents African taxa, light grey the Australasian taxa, and dark grey the Eurasian ones.

Estimated divergence times are indicated on the ML topology in Figure 2. A detailed chronogram is provided in the additional file 3. The standard deviations of all estimates fall between 0.5 to 0.7 Million years (My); this error value is implied in all divergence estimates indicated below. Divergence time estimations, standard deviations and credibility intervals calculated by multidivtime for the main nodes are indicated in the additional file 4, both for the combined dataset and for each gene separately. There is good congruence between the various estimations but with larger standard deviations for the ones based on one gene than for the values obtained with the combined dataset.

The earliest cladogenic event (to Lineage 1) is dated to 12.3 Mya. Emergence of the Clade C containing all African taxa as well as many Eurasian lineages is dated 11.1 Mya. Cladogenesis of the Afro-Asian Clades A and B is dated to 11 Mya. Divergences between each of Lineages 4 + 5, 6 + 7 and Clade B all fall within the interval 10.1–10.3 Mya. However, while these lineages originated more or less simultaneously, their subsequent diversification was unbalanced and asynchronous. Five of the seven lineages comprise only one or two genera (Lineages 4, 6, 7, 8 and 9), while the two most diverse and well-sampled lineages, corresponding to the main part of the African diversity, radiated somewhat at different times, at about 8.4 Mya (Lineage 10: 'arvicanthines') and 7.6 Mya (Lineage 5: '*Praomys *group'), respectively. As we have a good sampling within these African groups (14 of 18 genera in the 'arvicanthines' and 8 of 9 genera in the *Praomys *group), we are confident that our results accurately reflect the diversification histories of these lineages.

The phylogeny shows strong geographic structure (shown Figure 2) with most primary lineages restricted to a single biogeographic area. Notable exceptions are the genus *Mus *(Lineage 4), which includes both Eurasian and African sub-lineages, Lineages 9+10 which are predominantly African ('otomyines' and 'arvicanthines') but also includes the Asian genus *Golunda*, and the African *Praomys *group (Lineage 5) which also includes the Arabian species *Myomyscus yemeni*.

Three near-basal cladogenic events within Murinae correspond to separations between 'mostly Asian' and 'mostly African' lineages. The first of these, dated to 10.22 Mya, separates the *Praomys *group (Lineage 5) from the predominantly Asian genus *Mus*. The second, dated to 10.20 Mya, separates the African 'arvicanthines+otomyines' (Lineages 9+10) from the Asian *Millardia*/*Cremnomys *(Lineage 8). The third one, dated to 10.16 Mya, separates *Malacomys *from *Apodemus/Tokudaia*.

Within Lineage 10, there is a younger separation, dated to around 8.4 Mya, between the African 'arvicanthines' and *Golunda*, a genus currently found only in Asia. Within *Mus*, divergence of the African subgenus *Nannomys *from various Eurasian subgenera is dated to 6.6 Mya.

## Discussion

### Phylogenetic relationships of African Murinae and a new suprageneric taxonomy

Many of our ten primary lineages of Murinae were also identified by other scholars in previous molecular phylogenetic studies of Murinae [13,14,16,23,24,32]. However, our enlarged taxon sampling has improved the support for some relationships, which were tentatively identified in previous studies and also identified new primary lineages and associations. Based on these robust results and on the geographical structure of the phylogeny, we propose to formalize a tribal level of classification within Murinae (see Table 2), for convenient use above the informal rank of division employed by Musser and Carleton [8].

**Table 2:** Proposed tribal arrangment of the Murinae.

Tribes	Taxa in this study	Divisions	Additional taxa
		Musser and Carleton [8]	Musser and Carleton [8]
Arvicanthini	*Aethomys*	*Aethomys *division	
	*Micaelamys*		
	
	*Arvicanthis*	*Arvicanthis *division			
	*Desmomys*		
	*Lemniscomys*		
	*Mylomys*		
	*Pelomys*		
	*Rhabdomys*		
	
	*Dasymys*	*Dasymys *division	
	
	*Golunda*	*Golunda *division	
	
	*Hybomys*	*Hybomys *division	*Dephomys*
	*Stochomys*		
	
	*Grammomys*	*Oenomys *division	*Lamottemys*
	*Oenomys*		† *Malpaisomys*
			*Thallomys*
			*Thamnomys*

Otomyini	*Otomys*	Otomyinae	*Myotomys*
	*Parotomys*		

Millardini	*Cremnomys*	*Millardia *division	*Diomys*
	*Millardia*		*Madromys*

Apodemini	*Apodemus*	*Apodemus *division	† *Rhagamys*
	*Tokudaia*		

Malacomyini	*Malacomys*	*Malacomys *division	

Praomyini	*Colomys*	*Colomys *division	*Nilopegamys*
	*Zelotomys*		
	
	*Heimyscus*	*Stenocephalemys *division			
	*Hylomyscus*		
	*Mastomys*		
	*Myomyscus*		
	*Praomys*		
	*Stenocephalemys*		

Murini	*Mus*	*Mus *division	*Muriculus*

Hydromyini	*Apomys*	*Chrotomys *division	
	*Archboldomys*		
	*Chrotomys*		
	*Rhynchomys*		
	
	*Hydromys*	*Hydromys *division	*Crossomys*
			*Microhydromys*
			*Parahydromys*
			*Paraleptomys*
	
	*Conilurus*	*Pseudomys *division	*Leggadina*
	*Pseudomys*		*Leporillus*
			*Mastacomys*
			*Mesembriomys*
			*Notomys*
			*Zyzomys*
	
		*Pogonomys *division	*Abeomelomys*
			*Anisomys*
			*Chiruromys*
			*Coccymys*
			*Coryphomys*
			*Hyomys*
			*Macruromys*
			*Mallomys*
			*Mammelomys*
			*Pogonomelomys*
			*Pogonomys*
			*Spelaeomys*
			*Xenuromys*
	
		*Uromys *division	*Melomys*
			*Paramelomys*
			*Protochromys*
			*Solomys*
			*Uromys*
	
		*Xeromys *division	*Leptomys*
			*Pseudohydromys*
			*Xeromys*
	
		*Lorentzimys *division	*Lorentzimys*

Rattini		*Crunomys *division	*Crunomys*
			*Sommeromys*
	
	*Leopoldamys*	*Dacnomys *division	*Anonymomys*
	*Niviventer*		*Chiromyscus*
			*Dacnomys*
	
	*Maxomys*	*Maxomys *division	
	
	*Micromys*	*Micromys *division	*Chiropodomys*
			*Haeromys*
			*Hapalomys*
			*Vandeleuria*
			*Vernaya*
	
	*Bandicota*	*Rattus *division	*Abditomys*
	*Berylmys*		*Bullimus*
	*Bunomys*		*Kadarsanomys*
	*Diplothrix*		*Komodomys*
	*Rattus*		*Limnomys*
	*Sundamys*		*Nesokia*
			*Nesoromys*
			*Palawanomys*
			*Papagomys*
			*Paruromys*
			*Paulamys*
			*Taeromys*
			*Tarsomys*
			*Tryphomys*
	
		*Melasmothrix *division	*Melasmothrix*
			*Tateomys*
	
Phloeomyini	*Batomys*	*Phloeomys *division	*Carpomys*
	*Phloeomys*		*Crateromys*

Murinae *incertae sedis*		*Echiothrix *division	*Echiothrix*
	
		*Hadromys *division	*Hadromys*
	
		*Pithecheir *division	*Eropeplus*
			*Lenomys*
			*Lenothrix*
			*Margaretamys*
			*Pithecheir*
			*Pithecheirops*

The "divisions" of Musser and Carleton [8] are indicated as well as taxa not included in our analyses. Taxa for which there is independent molecular or morphological evidence of phylogenetic position are underlined (see text for details). All the other ones should be treated as Murinae incertae sedis. † : fossil genus.

Tribe Phloemyini (Lineage 1): A basal division within Murinae between certain Philippine 'Old Endemics' and all other murines was first suggested by Watts and Baverstock [10] based on microcomplement fixation of albumin, and strongly supported since then by numerous nuclear and/or mitochondrial gene phylogenies ([6,7,14,16,24], this study). Broader membership of this group includes two other endemic Philippine murine genera, *Carpomys *and *Crateromys *[14]. All members of this group are morphologically specialised in different ways but they do share at least one clearly derived dental trait – an unusually complex anteroconid morphology on the first lower molar [33]. The name Phloeomyinae Alston, 1876 (used at tribal level by Tullberg [34]) is available for this lineage. Musser and Carleton [8] recognised the same group as their *Phloeomys *division. We propose the tribe Phloeomyini Alston, 1876 new rank, for a clade containing the extant genera: *Batomys*, *Carpomys*, *Crateromys *and *Phloeomys*.

Tribe Rattini (Lineage 2): Our Lineage 2 corresponds in part to the 'South-East Asian clade' of Watts and Baverstock [10], the '*Rattus *group *sensu lato*' of Verneau et al. [31] and the '*Rattus *group' of Steppan et al. [24]. Jansa et al. [14] also recovered an equivalent lineage that includes various Philippines murines including *Crunomys *and members of the 'New Endemic' assemblage of Musser and Heaney [33]. Where our findings differ from most previous phylogenies is in the identification of the Eurasian harvest mouse, *Micromys minutus*, as the probable sister lineage to the '*Rattus *group *sensu lato*'. Previous results for *Micromys *either identified it as a basal lineage within Murinae [10,17,35], or hinted at a possible relationship with *Apodemus*, *Mus*, *Rattus *or *Tokudaia *[6,12,36-38]. Our conclusion that *Micromys *is linked to '*Rattus *group *sensu lato*' is also supported by the multilocus studies of Michaux et al. [32] and Rowe et al. [16]. Musser and Carleton [8] partitioned members of our Lineage 2 among five divisions (Table 2: *Crunomys*, *Dacnomys*, *Maxomys, Micromys *and *Rattus *divisions). Their *Micromys *division included five other Asian genera of arboreal murines (*Chiropodomys*, *Haeromys*, *Hapalomys*, *Vandeleuria *and *Vernaya*). Watts and Baverstock [10] identified a possible link between *Vandeleuria *and *Micromys *within Murinae, based on microcomplement fixation of albumin. However, Rowe et al.'s [16] recent multilocus molecular phylogeny of Murinae shows *Chiropodomys *as a sister lineage to our lineage 3, while *Vandeleuria *is a primary lineage within our clade A. Rowe et al. [16] also provide strong molecular evidence for the inclusion of genera *Melasmothrix*, *Chiromyscus*, and *Paruromys *into the clade that we here recognize as lineage 2. The only family level name that is based on a member of this group is Rattidae Burnett, 1830. This name is here applied for Lineage 2 at tribal level, as Rattini Burnett, 1830 new rank. Pending their inclusion in future molecular studies, we recommend that *Haeromys*, *Hapalomys *and *Vernaya *be treated as Murinae *incertae sedis*.

Tribe Hydromyini (Lineage 3): Our Lineage 3 corresponds to the 'Australasian group' identified by Steppan et al. [24]. Jansa et al. [14] recovered a clade that includes the Philippine members of this group but their study did not include any Australo-Papuan murines. Ford [15], using a combination of mitochondrial and nuclear intron sequences, demonstrated the close affinity of all Australian murine genera (*Rattus *excluded) but did not include any Philippine taxon in his study. Watts and Baverstock [10] included the majority of Australian and New Guinean murine genera in their microcomplement fixation study of albumin but they had poor coverage of Philippine murines. They failed to recover a single lineage that includes all Australo-Papuan murines. Studies of sperm ultrastructure also point to monophyly of the majority of Australo-Papuan murines, albeit with some notable exceptions [39,40]. Rowe et al. [16] included a wide array of Australo-Papuan and Philippine murines in their multilocus analysis, including representatives of the four suprageneric taxa recognised in previous studies of these regional faunas (i.e. uromyines, conilurines, hydromyines and anisomyines). Their results further confirm monophyly of the clade that we here define as tribe Hydromyini, and their study identifies *Chiropodomys *as the sister taxon of Hydromyini. Numerous family level names have been applied to members of our Lineage 3 (e.g. Hydromyina Gray, 1825; Coniluridae Dahl, 1897; Rhynchomyinae Thomas, 1897; Anisomyes Ellerman, 1941; Pseudomyinae Simpson, 1961; Uromyini Lee, Baverstock, and Watts, 1981). We recommend use of the name Hydromyina Gray, 1825 for this group, applied at tribal level as Hydromyini. Our application of this name is more inclusive than any prior usage, e.g. [39,41-44], and as group membership demonstrably includes each of *Conilurus*, *Pseudomys*, *Uromys*, *Anisomys *and *Rhynchomys *([24], this study), all of the other family level names based on Australasian murines either are objective synonyms of tribe Hydromyini or else are applicable only below this rank. We further recommend, pending further studies, that a suite of poorly studied Papuan genera be treated as *incertae sedis *within Murinae (Table 2). Use of one tribal name – Hydromyini – for this expended Australo-Papuan and Philippine murine radiation serves to draw attention to the phylogenetic connection between these geographically isolated assemblages. Musser and Carleton [8] divided members of our tribe Hydromyini among seven divisions (Table 2: *Chrotomys*, *Hydromys*, *Pogonomys*, *Pseudomys*, *Uromys*, *Xeromys *and *Lorentzimys *divisions).

Our Clade C contains a highly heterogeneous and geographically disparate assemblage, including all the African murines. Although this lineage has a poor basal support, a comparable assemblage was recently recovered with strength by Rowe et al[16], whose study clearly indicates that *Vandeleuria *also belongs to that clade. Within this group, we identify a total of seven primary lineages (Lineages 4–10), each well supported and geographically unified; and we note that the same seven lineages were recovered by Rowe et al. [16]. Our division of Clade C into two major sections [Clades A (Lineages 4–7) and B (Lineages 8–10)] is also supported by the results of previous multi-gene analyses [16,24], and by the presence of diagnostic indel events in the GHR alignment for several nodes (basal for Clade A; basal for Lineage 5), and we are confident as to the essential correctness of the topology. In terms of taxonomy, we might assign all members of Clade C to a single tribe, for which the earliest available name would be Murina Illiger, 1811. However, we prefer a more expansive tribal classification that recognises the huge taxic and ecomorphological diversity contained within Clade C. Accordingly, we propose to represent a total of seven tribes for each of Lineages 4–10. The result is an overall tribal classification of Murinae that is concordant in large measure with geographic partitioning and also has strong morphological expression.

Tribe Murini (Lineage 4): Our suggestion that the genus *Mus *be separated at tribal level is consistent with the previous lack of agreement over the sister taxon of this biomedically important genus [6,18,19,24,25]. As indicated above, the name Murina Illiger, 1811 is available and appropriate, adapted as tribe Murini (first used at this rank by Winge [42]). The position of African subgenus *Nannomys *within *Mus *is variously proposed to be polytomous with the other three subgenera of *Mus *[19,45], basal within *Mus *([20], this study), or as sister to the subgenus *Mus *[21]. However, a recent phylogenomic analysis gives compelling evidence that subgenus *Nannomys *is the second lineage to diverge within *Mus*, after subgenus *Coelomys *[46]. Musser and Carleton [8] recognised a *Mus *division and included *Muriculus *as a second genus. This rare African monotypic genus, endemic to Ethiopia, has not been available for molecular study. Osgood [47] noticed morphological links to *Mus *and to *Zelotomys*, a taxon here included within Lineage 5. Pending its inclusion in future molecular studies, we recommend that *Muriculus *be treated as Murinae *incertae sedis*.

Tribe Praomyini (Lineage 5): Our results agree with those of Steppan et al. [24] and Rowe et al. [16] on the identification of a diverse but almost exclusively African lineage as the sister lineage to *Mus*. Monophyly of this group (our Lineage 5) has strong nodal support and is further supported by a shared insertion in the GHR gene alignment. Lineage 5 corresponds to the '*Praomys *group' of Lecompte et al. [13,25,48]. We propose the new name Praomyini tribe nov. for this well-supported monophyletic assemblage, with *Praomys *Thomas, 1915 as type genus on account of its familiarity. Our results and those of previous studies [13,25,48], confirm inclusion within the Praomyini of *Colomys*, *Heimyscus*, *Hylomyscus*, *Mastomys*, *Myomyscus*, *Praomys*, *Stenocephalemys*, and *Zelotomys*. The genus *Nilopegamys*, previously considered as a subgenus of *Colomys*, is here regarded as a member of tribe Praomyini on morphological criteria [13]. Musser and Carleton [8] placed the members of our Praomyini in two divisions, the *Stenocephalemys *and *Colomys *divisions, based upon morphological and previous molecular datasets. Our results suggest a different arrangement of taxa within this group, with *Myomyscus verrauxii*, the type species of this problematic genus, grouping with *Colomys *and *Zelotomys *rather than with *Stenocephalemys *as suggested by Musser and Carleton [8]. Our expanded molecular dataset supports previous suggestions by Lecompte et al. [13,25,48], that each of *Myomyscus *and *Mastomys *are paraphyletic within the Praomyini. As in previous molecular and morphological analyses [13], the genus *Praomys *appears to be monophyletic with inclusion of *P. verschureni *and *P. daltoni*, although support is still quite low. Our enlarged dataset also resolves some relationships within the Praomyini, especially at the base of the clade, where resolution was poor in previous analyses [13]. The first lineage to diverge appears to be the clade *Heimyscus-Hylomyscus-Mastomys pernanus*, followed by the cluster *Myomyscus verreauxii*, *Zelotomys *and *Colomys*. The remaining members of this group (*Praomys*, all savanah-dwelling *Mastomys *except *M. pernanus*, *Stenocephalemys, Myomyscus brockmani *and *M. yemeni*) form a poorly supported cluster. However, within this cluster, one well supported sister-group relationship links the East African species *Myomyscus brockmani *and the Arabian species *Myomyscus yemeni*. Analysis of a larger suite of genes is necessary to clarify relationships within this interesting assemblage of African murines.

Tribe Malacomyini (Lineage 6): *Malacomys *has long been regarded as an isolated and enigmatic genus, whether assessed on dental morphology ([9]: 106) or on chromosomes [49]. Its isolated position is confirmed by our results and other molecular multilocus analyses [16,32]. The taxon Malacomyini tribe nov. is based on type genus *Malacomys *Milne-Edwards, 1877.

Tribe Apodemini (Lineage 7): *Apodemus *is among the most thoroughly studied of all murine genera, both from a molecular perspective, e.g. [17,38,50], and based upon the rich fossil record of western Eurasia, e.g. [51,52]. A close relationship between *Apodemus *and *Tokudaia *was suggested on dental morphology, e.g. [9], but molecular supporting data were only recently obtained [16,17,38]. Our analysis confirms a sister relation between *Apodemus *and *Tokudaia *but also highlight the considerable antiquity of their generic divergence. The taxon Apodemini tribe nov. is based on type genus *Apodemus *Kaup, 1829.

Our analysis identifies *Malacomys *as a possible sister lineage to *Apodemus *+ *Tokudaia*. Although nodal support is rather poor (77% BP; 0.69 PP) on our tree, we note that a comparable grouping of these lineages was observed in various other multi-locus analyses [13,16,32]. An exception is the multi-gene topology of Steppan et al. [24] in which *Malacomys *occupies a more basal position within a group corresponding to our Clade A. Musser and Carleton [8] recognised separate *Apodemus *and *Malacomys *divisions and we follow their lead in treating each of Lineages 6 and 7 as separate murine tribes. Moreover, since no included genus has previously formed the basis of a family level name, we propose two new names at tribal rank for these lineages. Although both lineages have limited generic diversity, we note that the genus *Apodemus*, despite being morphologically conservative, contains far greater molecular diversity than many other murine genera. Musser and Carleton [8] included the recently extinct genus *Rhagamys *from Corsica and Sardinia in the *Apodemus *division, based on paleontological interpretations of its dental morphology, e.g. [52], and we follow this lead.

All remaining murines examined in this study fall into our Clade B. Key members of this group are the Indian *Millardia *+ *Cremnomys *and the African 'arvicanthines' and 'otomyines'. Phyletic association of *Otomys *+ *Parotomys *with the 'arvicanthines' is robustly supported by numerous other molecular analyses and must now be considered as proven [6,11,12,14,16,24]. Association of *Millardia *+ *Cremnomys *with this group is a more controversial finding, although we note a comparable topology in the DNA/DNA hybridization results of Chevret [23] and partial support from several recent molecular analysis [16,32]. Ducroz et al. ([12]: p 200) found no evidence from analyses of mitochondrial DNA of close relationship between *Millardia *and African arvicanthines, while Watts and Baverstock ([10]: p111) concluded from their albumin immunology that "*Millardia *appears to be a monogeneric lineage arising early in the history of the murines". Rowe et al. [16] identified conflict among the three genes available for the position of *Millardia*. Our analysis differs mainly in the inclusion of two *Millardia *species and a representative of the genus *Cremnomys *and this wider taxon sampling may account for the improved support for the sister group relationship of this lineage with the 'arvicanthines' and 'otomyines'. However, conflict with previous analysis highlights the need for further testing of this relationship using sequences from other slowly evolving nuclear genes.

Consistent with our treatment of Clade A, we propose to recognize three separate tribes within Clade B, an arrangement that in our view best reflects the taxic and morphological diversity, and the geographic partitioning of this assemblage.

Tribe Millardini (Lineage 8): We propose to recognize as tribe the predominantly Indian genera *Millardia *and *Cremnomys*. Musser and Carleton [8] distinguished this lineage as their *Millardia *division. We propose Millardini tribe nov., with type genus *Millardia *Thomas, 1911 and referred genus *Cremnomys*.

Tribe Otomyini (Lineage 9): Traditional recognition of a subfamily Otomyinae for the African genera *Otomys *and *Parotomys *reflects the extreme specialization of the cheekteeth of these taxa, especially among members of the genus *Otomys*. Despite compelling molecular [6,11,12], and paleontological [53-55] evidence that otomyines not only belong within Murinae but are specifically associated with arvicanthines ([14,16,24], this study), the notion of taxonomic isolation maintains an inertia that is difficult to break, e.g. Musser and Carleton [8]. Like some previous authors [55,56], we advocate recognition of this lineage at tribal level, as Otomyini Thomas, 1896 with type genus *Otomys *Cuvier, 1824.

Tribe Arvicanthini (Lineage 10): Ducroz et al. [12] proposed a tribe Arvicanthini but failed to explicitly designate a type genus. As indicated by Musser and Carleton [8], their name is a *nomen nudum *and nomenclaturally unavailable. We here formalise the Arvicanthini tribe nov. with type genus *Arvicanthis *Lesson, 1842. The tribe corresponds in large part to Misonne [9] 's '*Arvicanthis *division' but with notable additions (*Oenomys*, [11,12,24], this study) and exceptions (*Bandicota *and *Nesokia*, both close relatives of *Rattus*, [32,57], this study). The arvicanthine affinity of the Indian genus *Golunda *was promoted on dental criteria by each of Misonne [9] and Musser [58], and was weakly supported by the 12S and 16S mitochondrial gene phylogenies of Ducroz et al. [12] and by the IRBP and cytochrome b phylogeny of Michaux et al. [32]. Our results confirm this association, with moderately strong nodal support, and provide, for the first time, a basal position for *Golunda *within the tribe. Based on earlier molecular work and our expanded taxon sampling, confirmed members of the tribe Arvicanthini are *Aethomys*, *Arvicanthis, Dasymys*, *Desmomys*, *Golunda*, *Grammomys*, *Hybomys*, *Lemniscomys, Micaelamys, Mylomys, Oenomys*, *Pelomys, Rhabdomys*, *Stochomys, Thallomys *and *Thamnomys *([12,16,24,32], this study). Our tribe Arvicanthini thus includes genera of the *Aethomys*, *Arvicanthis*, *Dasymys*, *Golunda*, *Hybomys *and *Oenomys *divisions of Musser and Carleton [8] (see Table 2). Our phylogeny for Arvicanthini is the first one based on nuclear genes and it also features enlarged taxon sampling. We confirm earlier mtDNA evidence [12] of a clade containing *Arvicanthis, Desmomys*, *Lemniscomys, Mylomys, Pelomys*, and *Rhabdomys*, and for sister-group relationships between *Mylomys *and *Pelomys*, and between *Desmomys *and *Rhabdomys*. Our results depart from previous interpretations in the well-supported grouping of *Arvicanthis *and *Lemniscomys *as sister taxa (*Lemniscomys *occupied a basal position within the clade in previous analyses [12]). The inclusion of previously unsampled taxa in our phylogeny also produced new insights into Arvicanthini phylogeny, most notably the basal position of *Golunda*, followed by the divergence of *Oenomys *then by the highly supported clade containing *Stochomys *and *Hybomys*. The basal position of *Oenomys *among the arvicanthini was also proposed in a recent molecular study [16] despite sparse sampling within the tribe. The other associations identified here are not supported by previous analyses and they require further testing with sequences from other slowly evolving nuclear genes.

Some genera, not yet available for molecular phylogenetic studies, can be associated with the Arvicanthini on morphological criteria. For example, the rare African genus *Dephomys *shares dental and cranial morphometric traits with *Hybomys *[9,59], and was included in the *Hybomys *division by Musser and Carleton [8]. Similarly, the monotypic genus *Lamottemys*, described after the work of Misonne, is thought be closely related to *Oenomys *[60,61], and was included in the *Oenomys *division by Musser and Carleton [8]. *Malpaisomys*, an extinct genus from the Canary Islands, was also included in the *Oenomys *division by Musser and Carleton [8], based on morphological studies by Lopez-Martinez et al. [62] and their own assessment. These authors also suggest that *Canariomys*, the other murine endemic from the Canary Island, might be a member of this divison but that morphological reexamination of the specimens is needed. Finally, the Manipur bush rat, genus *Hadromys*, was included within the *Arvicanthis *division by Misonne [9] but regarded as potentially distinct from this lineage by Musser [58]. Musser and Carleton [8] placed this Indian genus in its own monotypic division and we follow suite by listing it as *incertae sedis *within Murinae (Table 2).

### Timing of cladogenesis among African lineages

Several authors have estimated divergence times among muroids from molecular data [7,11,12,14,16-18,38,63]. These studies have involved different gene and taxon sampling, and used a variety of different methods and means of calibration. Not surprisingly, the results are quite variable. Our estimates for the timing of key cladogenic events for the African murine diversity, based on a relaxed molecular clock, are: 10.2 Mya (± 0.6) for origin of Arvicanthini+Otomyini; 10.2 Mya (± 0.6) for origin of Praomyini; 10.2 (± 0.5) for the origin of *Malacomys*; 8.4 My (± 0.6) for the origin of extant arvicanthine lineages; 7.6 My (± 0.6) for the origin of extant Praomyini; and 6.6 Mya (± 0.7) for the origin of extant subgenera within *Mus *including the African subgenus *Nannomys *(Figure 2). Our estimates for the timing of other cladogenic events are presented in additional file 3 and 4.

Our divergence time estimates are consistently older than those calculated by Chevret et al. [11,63], based on a DNA hybridization dataset. The differences reflect their use of a different calibration (10 My for *Mus*/*Rattus *divergence) combined with a fixed-rate molecular clock. Our estimates for origin of extant arvicanthine and praomyine lineages are consistent with the 8 My estimate obtained by Ducroz et al [12] for arvicanthines but younger than the 8.5 Mya estimate for Praomyini obtained by Lecompte et al. [25]. Both studies used mitochondrial DNA sequences, the same calibration points (*Mus*/*Rattus *divergence at 12 Mya and/or Murinae/Gerbillinae divergence at 16 Mya), and a fixed-rate molecular clock. In a more recent paper using a combined cyt *b *and IRBP dataset and a *Mus*/*Rattus *divergence time set to 12 Mya, Lecompte et al. [13] derived estimates of 7.4–9.3 Mya for the origin of extant lineages with Arvicanthini and 6.7–8.4 Mya for lineages within Praomyini, results that are congruent with those reported here.

Steppan et al. [7] derived divergence estimates from a four nuclear gene concatenation, using a variety of different estimation methods and a 12 My fossil calibration point for the basal radiation of all extant Murinae. Since their study included *Batomys*, a member of our Phloeomyini, this represents a deeper divergence than the usual *Mus/Rattus *split assigned to 12 Mya. Their divergence estimates (8.8–10.3 Mya for *Mus*/*Rattus*, 7.9–9.7 Mya for *Mus*/*Arvicanthis *and 6.9–8.8 My for *Mus*/*Mastomys*) are consistently younger by 1–2 Mya than those obtained here. A similar difference in estimates of divergence times is observed between the multilocus study of Rowe et al. [16] and our results (for example, Mus/Rattus at 9.7 ± 0.5 versus 11.3 ± 0.5 Mya). As rightly pointed by Steppan et al. [7] and Rowe et al. [16], these differences most obviously reflect the nodal assignment on the topology of the crucial transition from fossil *Antemus *to fossil *Progonomys *at 12.1 Mya. In addition, the differences may also reflect selection of other calibration points, and the differences in taxon sampling.

Several molecular studies on *Apodemus *suggest an early divergence between *Tokudaia *and *Apodemus *as well as between the main lineages within *Apodemus *[17,37,38,50]. We derived estimates of 10.2 Mya (± 0.5) for the separation of *Apodemus *and *Malacomys*, 9.6 (± 0.5) for *Apodemus*/*Tokudaia *and 8.6 (± 0.5) for the earliest divergence within *Apodemus*. Similar estimates were found by Michaux et al. [17] but Sato and Suzuki [38] obtained highly variable times for the *Apodemus*/*Tokudaia *divergence with each of their five data sets, ranging from 6.5–7.6 My for IRBP to 11.3–13.2 Mya for mitochondrial cyt *b*.

The genus *Mus *has been subjected to extensive phylogenetic study, e.g. [18,20,45], though in most studies the African *Nannomys *was underrepresented. We estimated the initial divergence of extant *Mus *[including *Nannomys*] lineages to 6.6 Mya (± 0.7), with *Nannomys *as the earliest offshoot. Catzeflis and Denys [19] dated the divergence between *Nannomys *and other *Mus *subgenera to between 5.7 and 4.7 Mya, based on the DNA hybridization method and a 10 Mya calibration point for the *Mus*/*Rattus *divergence. Subsequently, Chevret et al. [64] used a 12 Mya calibration point for the *Mus*/*Rattus *divergence and revised the *Nannomys *divergence to 5.7 Mya and that of *Coelomys *to 6.5 Mya. By also using a calibration point set at 12 My for the *Mus/Rattus *split, other studies suggested younger (5.1 to 5.2 Mya: Suzuki et al. [18]) or similar (6.8 to 7.8 Mya: Chevret et al. [20]; 7.6 ± 1.1 Mya: Veyrunes et al. [21]) timing for the initial divergence of subgenera within the genus *Mus *(inclusive of *Nannomys*).

Jansa et al. [14] presented divergence time estimates for murines that are considerably older than our own. For example, based on IRBP sequences they estimated the divergence date between our Hydromyini and our Murini+Praomyini+Arvicanthini at 15.8–20.5 Mya, depending on calculation method used. These values are much older than our estimate of 11.1 ± 0.5 Mya for this divergence. We suspect that Jansa et al. [14] systematically overestimated divergence times within Murinae through their use of fossil calibration points placed on more basal nodes in the Rodentia as well as in the general mammalian tree, leading to an increased likelihood of partial saturation at mutational hotspots. Jansa et al. [14] defended their divergence estimates by referring to the incompleteness of the fossil record, especially the fact that large parts of the Old World have almost no relevant small mammal fossil record.

To further explore this conflict in interpretation, we tested our molecular divergence framework within the Murinae against the relatively good fossil record of this group in Europe, Africa, and South Asia. As shown on Figure 2, the earliest first fossil occurrences of various lineages all fall within the time ranges suggested by our divergence date estimates. Moreover, we note that the oldest fossil Murinae from South Asia and Africa, estimated to be about 12–14 Mya and 10–11 Mya, respectively (Asia: [65,66]; Africa: [67-71]) are not attributable to extant genera (e.g. *Progonomys*: [72]; *Karnimata*: [70,73]); or only tentatively so (c.f. *Stenocephalemys*, c.f. *Parapelomys*: [71]; c.f. *Lemniscomys*: [74,73]). Conversely, representatives of modern genera are not definitely recorded prior to 5–7 Mya [73,75-77] which is consistent with our dating of murine evolution but difficult to reconcile with a much longer evolutionary time frame. Even more convincingly, our divergence estimates are consistent with first appearance of murines in the fossil records of Africa around 12 Mya [30,71,72] and in Europe around 11 Mya [78,79].

### Biogeographic implications for African murines

Our molecular phylogeny contributes in several ways to an improved understanding of the pattern and timing of initial murine colonization of Africa. The earliest, generally accepted murine fossils occur in the sedimentary record of the Siwalik Hills of Pakistan, and date to around 14 Mya [65,66,80,81]. In contrast, the earliest murine fossils from anywhere in Africa date to less than 12 Mya [68], despite the fact that other groups of muroid rodents (including the genus *Potwarmus*, a taxon of uncertain subfamilial affinity) are represented in older fossil deposits, e.g. [69,71,82]. Similarly, the abundant fossil record of Europe contains no evidence of murines prior to 11 Mya, at which time they appear fully differentiated and undergo rapid diversification [78,79]. This disparity between the various regional fossil records suggests that Murinae originated in Asia and colonized both Africa and Europe during a common period of dispersal [30,72]. Our molecular phylogeny of Murinae is consistent with this scenario to the extent that each of the three basal branches on our phylogeny (Phloeomyini, Rattini and Hydromyini) is almost entirely restricted to Asia and/or the major islands of the western Pacific (i.e. Philippines and Australasia). The major exceptions are *Micromys*, an extant genus with a wide Palearctic distribution [8] but with no known African fossil record [83], and the fossil genus *Karnimata*, which is best known from the Siwalik sequence but is also reported from late Miocene localities in southern and eastern Africa [77]. *Karnimata *is a possible stem genus for our Rattini [65,72], and its presence in Africa, if confirmed by further study of the fossils, would imply that some early immigrant lineages died out without leaving modern descendants.

Jacobs et al. [80] postulated that dispersal of murines from Asia to Africa started around 11.8 Mya, following establishment of a vegetation corridor between Africa and Asia across the recently established Arabian peninsula [30,76,84-87]. The best evidence of intercontinental dispersal by mammals during this period is the sudden appearance of equids ('*Hipparion*') in the African fossil record [86,88,89]. Significantly, the earliest African hipparionines and murines occur together in sites dated to around 11 Mya in Algeria [68] and 10 Mya in Ethiopia [86,90]. Just how many murine lineages crossed from Eurasia into Africa during this early period of dispersal is less certain, with somewhat contradictory indications coming from each of the fossil record and the molecular phylogeny.

The earliest fossil murines from African localities are referred to the genus *Progonomys *[68,86,90,91]. Slightly younger localities in Namibia and East-Africa, dated to around 9–10 Mya contain more diverse murine faunas with *Karnimata *sp., *Aethomys*, c.f. *Parapelomys *sp. and c.f. *Stenocephalemys *sp. [69-71,92]. As noted above, *Karnimata *is a typical Asian Miocene genus but the other taxa suggest an early period of *in situ *diversification leading to each of the endemic African praomyine and arvicanthine lineages. In apparent contradiction to this scenario, our molecular phylogeny suggests that each of three early branches of the African murine radiation (Praomyini, Arvicanthini+Otomyini and Malacomyini) has a sister lineage among Eurasian Murinae (Murini, Millardini and Apodemini, respectively). The obvious interpretation is that each of these lineages was differentiated prior to their dispersal into Africa, and arrived around the same time as part of a broader episode of faunal interchange. Our divergence time estimates would place this period of faunal interchange followed by regional differentiation in the interval 11–10 Mya – a very good fit with the fossil record of Africa and Asia. However, an alternative scenario, only marginally more complex, could posit an early dispersal to Africa, followed by differentiation and back dispersal of three lineages from Africa to Eurasia (ancestral Murini, Apodemini and Millardini). A detailed reassessment of the earliest African murine fossils, looking for evidence of phyletic continuity *versus *disjunction, might resolve this issue. Until this is done, we must be content with the notion of a shared biogeographic province spanning the 'Arabic Corridor' across which various early murines referrable to *Progonomys, Karnimata *and possibly other genera made their way between southwest Asia and northern Africa, starting around 11 Mya. These populations presumably included basal members of the Apodemini *+ *Malacomyini, the Murini *+ *Praomyini, and our Clade B (stem group of Millardini + Otomyini + Arvicanthini).

The earliest African fossil faunas of fully modern aspect (i.e. with species confidently assigned to extant genera) date to the interval 7–5 Mya [73,75-77,92-95]. However, due to sizable gaps in the African fossil record, it is currently unclear whether these later murines were derived from the earliest colonists or from a later wave of colonization from Asia, or perhaps from a combination of both. Certainly, the appearance around 7–9 Mya in the African record of distinctively Asian lineages of Bovidae [96], Elephantoidea [97] and non-murine rodents [30,76,98] is strong evidence for habitat continuity and dispersal between Asia and Africa during the terminal Miocene. However, the rise to dominance of the Gerbillinae in the fossil record of the Middle East during the interval 7–8 Mya also suggests increasingly arid conditions on the Arabian Peninsula [84,99]. This may have presented a barrier to dispersal by murine rodents, and and hence, caused the onset of independent diversification of the African and Asian murine faunas. Direct evidence for murine dispersal into Africa during this period is limited by the paucity of the fossil record.

We estimate the timing of diversification of modern Arvicanthini + Otomyini at 8.6 ± 0.6 Mya, and of modern Praomyini at 7.6 ± 0.6 Mya. Diversification of the modern African murine genera thus seems to narrowly postdate the disruption of the Arabic Corridor.

After 6 Mya, there is renewed evidence of faunal interchange between Africa and each of Southwest Asia and Western Europe [28,76,91,100-105]. This coincides with a period of global sea level depression [106], and with the combination of eustatic and tectonic events in the Mediterranean region that precipitated Messinian salinity crisis [84,107]. Fossil evidence from the circum-Mediterranean region through this period documents significant dispersal and associated mammalian turnover [28,84,100,102,108,109]. Among murine rodents, a species of *Mus *probably entered Africa from Asia around this time, somewhere between 6.6 ± 0.7 Mya (the divergence estimate for the subgenus *Nannomys *within *Mus*) and 4.0 ± 0.8 Mya (the earliest cladogenic event within subgenus *Nannomys *[20,21]). The earliest fossil occurrence of *Mus *in Africa comes from Kenya, dated to 4.5 Mya [76]. Around the same time, a species of *Myomyscus *(Praomyini) evidently spread to the Arabic region, giving rise to the modern species *M. yemeni*. We estimate the time of divergence of this species from its East African sister species (*M. brockmani*) at 5.1 ± 0.6 Mya, which also coincides locally with the opening of the Red Sea. In North Africa, the western European fossil genus *Occitanomys *is recorded for the first time in a section younger than 5.32 Mya [91]. Finally, the fossil record also provides some examples of late Tertiary murine dispersal between Asia and Africa. Most notably, African sites of latest Miocene-Pliocene age reportedly contain several 'Indian' genera (*Millardia *and *Golunda*) [91,98,110], while Asian localities of latest Miocene and early Pliocene age have produced several genera of possible arvicanthines. One such lineage is the extinct arvicanthine genus *Saidomys*, with a stratigraphic range that extends back to the late Miocene in Africa [76,104], to the early Pliocene in Pakistan and Afghanistan [28,100], and to the latest Pliocene in Thailand [111]. The extinct genus *Parapelomys*, known from several South Asian localities of latest Miocene and early Pliocene age, is also touted as possible arvicanthine [28,112].

Environmental changes after 3 Myr probably caused the regional extinction of some lineages and generally shaped the modern continental faunas [113-115]. The genera *Millardia *and *Golunda *may have disappeared from Africa, while *Saidomys *and *Occitanomys *went to global extinction. Over the same period, numerous groups of African murines radiated to fill newly emerging habitats. However, few were quite so successful as the African pigmy mice (18 living species are recognized for the subgenus *Nannomys *[8]), which appear to have found a largely underexploited set of niches below the body size range of other African murines.

## Conclusion

Our molecular dataset for Murinae, which includes the most complete sampling so far of the African murines, gives compelling evidence for five phyletically separate radiations within the African region, as well as several phases of dispersal between Asia and Africa during the late Miocene to early Pliocene. Through our expanded taxon sampling, which also includes a good coverage of Eurasian taxa we also reveal many new details concerning the overall phylogenetic structure of the Murinae, and this forms a basis for rational classification at tribal level of this traditionally problematic group. Further studies of Murinae should target the few remaining African genera that were not available in our dataset (including *Thallomys*, *Lamottemys *and *Muriculus*), as well as various unsampled Asian taxa (e.g. *Hapalomys*, *Lenothrix*) including those that have been associated with the African Arvicanthini on morphological grounds (e.g. *Hadromys*). Dense taxon sampling of the Australo-Papuan Hydromyini was recently provided by Rowe et al. [16], although a few important gaps remain for this region. On a broader level, a comparison of the phylogenetic structure of Murinae with that of other co-distributed groups of small mammals, such as Gerbillinae and Soricidae, might shed even greater light on the history of the faunal interchange and extinction across Africa and Asia during the last 15 My.

## Methods

### Taxon and gene sampling

We obtained sequences from 83 species including representatives of 49 murine genera from most previously identified major murine lineages, as well as eight genera of Deomyinae and Gerbillinae (Table 3) for use as outgroups [5-7]. Our sampling for African Murinae and otomyines covers 25 out of 32 living African genera and includes representatives of all the four previously identified lineages. Most genera are represented by a single species but multiple representatives are included for highly diversified or potentially paraphyletic genera.

**Table 3:** List of the taxa examined in this study and their GenBank accession numbers.

Famille	Taxa	**cyt *b***	IRBP	GHR
**Murinae**	*Aethomys chrysophilus*	AJ604515	AY326075	NA
	*Apodemus argenteus*	AB032848	AB032855	NA
	*Apodemus flavicollis*	AB032853	AB032860	AM910943*
	*Apodemus mystacinus*	AF159394	AJ311158	AM910942*
	*Apodemus speciosus*	AB032849	AB032856	NA
	*Apodemus sylvaticus*	AB033695	AB032863	NA
	*Apomys hylocoetes*	AY324467	NA	AY294915
	*Archboldomys luzonensi*	AY324460	DQ191495	NA
	*Arvicanthis niloticus*	AF004569	DQ022386*	AM910944*
	*Arvicanthis somalicus*	AF004573	NA	AY294918
	*Bandicota bengalensis*	AM408340	AM408331	AM910945*
	*Batomys granti*	AY324459	DQ191496	AY294917
	*Berylmys bowersii*	AM408337	AM407896	AM910946*
	*Bunomys chrysocomus*	AM910934*	AM910937*	AM910947*
	*Chrotomys gonzalensi*	AY324461	DQ191503	NA
	*Colomys goslingi*	AF518372	DQ022395 *	AM910948*
	*Conilurus penicilatus*	AM910935*	AM910938*	AM910949*
	*Cremnomys cutchicus*	DQ022381	DQ022384	NA
	*Dasymys incomtus*	AF141217	EU292143*	AM910950*
	*Desmomys harringtoni*	AF141206	EU292144*	NA
	*Diplothrix legata*	AB033696	AB033706	NA
	*Golunda ellioti*	AM408338	AM408332	AM910951*
	*Grammomys macmillani*	AM408345	AM408329	AM910980*
	*Grammomys sp*.	AF141218	DQ022389	AM910952*
	*Heimyscus fumosus*	AF518333	DQ022397*	AM910953*
	*Hybomys univittatus*	AF141219	DQ022388*	DQ019059
	*Hydromys chrysogaster*	AM408339	AM408319	AM910954*
	*Hylomyscus parvus*	AF518330	DQ022399	DQ019060
	*Hylomyscus stella*	AF518331	AM408320	AM910955*
	*Lemniscomys striatus*	AF141210	AM408321	AM910956*
	*Leopoldamys edwardsi*	AJ698881	AJ698897	NA
	*Malacomys edwardsi*	DQ022379	DQ022392*	AM910958*
	*Malacomys longipes*	AM408341	DQ022393*	AM910957*
	*Mastomys erythroleucus*	AF518338	AM408335	AM910959*
	*Mastomys natalensis*	AF518342	AY518342	NA
	*Mastomys pernanus*	AF518343	DQ022403*	AM910960*
	*Mastomys kollmannspergeri*	AF518345	DQ022402*	AM910961*
	*Maxomys whiteheadi*	EU292150*	AY326094	NA
	*Micaelamys namaquensis*	AF141215	AM408330	AY294914
	*Micromys minutus*	AB033697	AB033710	NA
	*Millardia kathleenae*	EU292148*	EU292145*	AM910963*
	*Millardia meltada*	AF141221	AM408322	AM910962*
	*Mus (Coelomys) crociduroides*	AJ698878	AJ698894	AM910964*
	*Mus (Nannomys) minutoides*	AY057816	AJ875086	NA
	*Mus (Mus) musculus*	V00711	AB033711	AY271378
	*Mus (Pyromys) platythrix*	AJ698880	AJ698895	NA
	*Mylomys dybowski*	AF141212	EU292146*	AM910965*
	*Myomyscus brockmani*	AF518353	DQ022407*	AM910966*
	*Myomyscus verreauxii*	AF518355	DQ022408*	AM910967*
	*Myomyscus yemeni*	AF518357	DQ022409*	AM910968*
	*Niviventer niviventer*	AM408344	AM408323	AM910969*
	*Oenomys hypoxanthus*	AM408342	AM408324	AM910970*
	*Otomys angoniensis*	AM408343	AM408325	AM910971*
	*Parotomys sp*.	NA	NA	AY294912
	*Pelomys fallax*	DQ022382	DQ022391	NA
	*Phloeomys cumingi*	DQ191484	AY326103	DQ019076
	*Praomys daltoni*	AF518349	DQ022406*	AM910972*
	*Praomys degraaffi*	AF518359	DQ022410	NA
	*Praomys jacksoni*	AF518361	AM408326	AM910973*
	*Praomys misonnei*	AF518364	DQ022412	NA
	*Praomys tullbergi*	AF518365	AM408327	AM910974*
	*Praomys verschureni*	AF518373	DQ022394*	NA
	*Pseudomys australis*	AM910936*	AM910939*	AM910975*
	*Rattus exulans*	DQ191486	AY326105	DQ019074
	*Rattus norvegicus*	VO1556	AB033714	J04811
	*Rattus rattus*	AB033702	AM408328	AM910976*
	*Rattus tanezumi*	AB096841	AB096856	NA
	*Rhabdomys pumilio*	AF141214	AY326106	AY294913
	*Rhynchomys isarogensis*	AY324462	AY326108	DQ019075
	*Stenocephalemys albipes*	AF518347	DQ022404	AM910977*
	*Stenocephalemys albocaudata*	AF518370	DQ022414*	AM910978*
	*Stochomys longicaudatus*	EU292149*	EU292147*	DQ019076
	*Sundamys muelleri*	AM408340	AY326111	AM910979*
	*Tokudaia osimensis*	AB029429	AB033712	AM910981*
	*Zelotomys hildegardeae*	AF518375	DQ022396*	DQ019080

**Deomyinae**	*Acomys*	AJ233953 (*cahirinus*)	AJ698898 (*cahirinus*)	AY294923 (*ignitus*)
	*Deomys ferrugineus*	NA	AY326084	AY294922
	*Lophuromys flavopunctatus*	AY828236	AY326091	AY294921

**Gerbillinae**	*Desmodillus auricularis*	AJ851272	AM910940*	DQ019048
	*Gerbillurus paeba*	AJ430557	AM910941*	NA
	*Gerbillus gerbillus*	AJ851269	NA	DQ019049
	*Meriones*	AF159405 (*unguiculatus*)	AY326095 (*unguiculatus*)	AF332021 (*shawi*)
	*Gerbilliscus robustus*	AJ875234	AY326113	AY294920

*: newly acquired sequences. NA: sequence not available.

Sequences were obtained for two single-copy nuclear genes (growth hormone receptor exon 10: GHR; and interphotoreceptor retinoid binding protein exon 1: IRBP) and one mitochondrial-coding gene (cytochrome *b *apoenzyme: cyt *b*). Specimen identification and sequence data are listed in Table 3.

The nuclear genes were chosen because of their proven utility for understanding muroid relationships and the presence of an existing sequence dataset for this group [6,7,14,24,116,117]. The GHR and IRBP genes are not genetically linked and their location is variable, on chromosomes 15 and 14 in *Mus*, and chromosomes 2 and 16 in *Rattus *[118]. The mitochondrial cytochrome *b *gene was chosen because it provides a third independent marker that evolves at a faster rate than either of the two nuclear genes, and also is well represented in previous datasets.

Most taxa are represented by sequences from two or three genes, the one exception being *Parotomys *for which we have only GHR sequence (Table 3). All ingroup genera are represented by sequences from the same species and where possible, by sequences from the same DNA sample. Chimeric data (i.e. different sequences deriving from more than one species of a genus) were used only for two outgroup taxa: *Acomys *(*A. cahirinus *and *A. ignitus*) and *Meriones *(*M. unguiculatus *and *M. shawi*).

### DNA extraction and sequencing

Total genomic DNA was extracted from tissues preserved in ethanol using a CTAB protocol [119] or a QiaAmp extraction kit (Qiagen). The cytochrome *b *(1140 bp) gene was amplified as described in Lecompte et al. [25] or Montgelard et al. [120]. PCRs used the following thermal cycling parameters: one step at 94°C for 4 min, followed by 35 cycles (40 s at 94°C, 45 s at 50°C, 1 min at 72°C). The final extension at the end of the profile was at 72°C for 10 min.

Part of exon 1 of IRBP (ca 1270 bp) was sequenced, using the methods of Poux and Douzery [121]. Amplification of the IRBP gene was performed under the same conditions: one cycle of 94°C denaturation (5 min), 50°C annealing (45 s), 72°C extension (1 min); 34 cycles of 94°C denaturation (45 s), 50°C (or 60°C) annealing (45 s), 72°C extension (1 min); and a final extension of 72°C (10 min).

Exon 10 of the GHR gene was amplified using the following parameters: 95°C (5 min); 5 cycles of 95°C (30 s), 61°C (30 s), 72°C (1 min); 5 cycles of 95°C (30 s), 59°C (30 s), 72°C (1 min); 5 cycles of 95°C (30 s), 57°C (30 s), 72°C (1 min), 5 cycles of 95°C (30 s), 55°C (30 s), 72°C (1 min); 20 cycles of 95°C (30 s), 53°C (30 s), 72°C (1 min); and a final extension of 72°C (10 min). The primers used were GHR 1 (= GHREXON10, [122]) and GHR2 (GATTTTGTTCAGTTGGTCTGTGCTCAC) and two internal primers GHR7 (AAGCTGATCTCTTGTGCCTTGACCAGAA) and GHR8 (TTGGCATCTGACTCACAGAAGTAGG).

Double-stranded PCR products were purified directly from the PCR product or from agarose gel using the MinElute purification kit (Qiagen) or Amicon Ultrafree-DNA columns (Millipore) and sequenced directly on both strands using an automatic sequencer CEQ2000 (Beckman) or an ABI 310 (PE Applied Biosystems).

The new sequences were deposited in the EMBL data bank. Accession numbers for all sequences used in this analysis are listed in Table 3.

### Analyses

#### Phylogenetic reconstruction

Sequences were manually aligned with the ED editor of the MUST package version 2000 [123]. Nonsequenced positions and gaps were coded as missing data. Phylogenetic reconstructions were performed on the complete DNA data set by maximum likelihood (ML) with PAUP* (version 4 beta 10) [124], and by Bayesian inference (BI) with MrBayes (version 3.1.2) [125].

Modeltest 3.7 [126] was used to determine the sequence evolution model that best fits our data using the Akaike Information Criterion (AIC). This program examined the fit of 56 models, with either a proportion of invariable sites (I), a gamma distribution of substitution rate variation among-sites (G), or a combination of both (I + G).

To avoid excessive calculation times, our PAUP* ML analyses were conducted in two steps. A ML heuristic search was first conducted by Tree Bisection Reconnection (TBR) branch swapping to identify the optimal tree under parameters estimated by Modeltest. This tree was re-used for a new round of parameter estimation/branch swapping. This procedure was repeated until there was a stabilization of both topologies and parameters. The robustness of nodes was estimated in PHYML [127] with ML bootstrap percentages (BP_ML_) estimated from 1000 pseudoreplicates using as a starting tree the best ML tree obtained from PAUP. PHYML was preferred over PAUP* for bootstrap analyses because of its rapidity. We also performed Bayesian Inference, as calculated by MrBayes, and report Posterior Probabilities (PP) for recovered nodes. For the Bayesian analysis we used 9 partitions, one for each codon position of each gene.

#### Estimating dates of divergences

Divergence times were estimated for the optimum ML topology. The hypothesis of a constant molecular clock was tested by a Likelihood Ratio Test as proposed by Felsenstein [128] and calculated in PAUP*4.0b10. We used a relaxed Bayesian molecular clock approach as implemented in MultiDivTime [129], using parameter estimates derived with PAML [130] as described by Yoder and Young [131]. Divergence times were estimated with two fossil-based calibration intervals: 1) the *Mus/Rattus *divergence set to between 10–12 Mya [65,66,132,133]; and 2) the divergence between *Apodemus mystacinus *and all the species of subgenus *Sylvaemus *(*A. flavicollis *and *A. sylvaticus*) set to a minimum of 7 Mya [51,78].

## Authors' contributions

EL and PC initiated the study and assembled the data. EL, KA, CD and FC collected specimens in the field and/or provided tissue samples. MC, EL and PC obtained sequences. PC ran the calculations. FC, KA and CD all contributed to improving the manuscript. All authors read and approved the manuscript.

## Supplementary Material

Additional file 1 **Maximum likelihood topology obtained with the combined dataset**. The support values from each gene separately are indicated for the main nodes discussed in the text. The support values are indicated as follow: GHR/IRBP/cytb. A black dot indicate that the node is supported by the three dataset with a BP > 95, +: BP > 95 otherwise the BP value is indicated, ø: no data available, -: not supported by the dataset. Click here for file

Additional file 2 **Bayesian topology obtained with the combined dataset**. The support values from each gene separately are indicated for the main nodes discussed in the text. The support values are indicated as follow: GHR/IRBP/cytb. A black dot indicate that the node is supported by the three dataset with a BP > 95, +: BP > 95 otherwise the BP value is indicated, ø: no data available, -: not supported by the dataset. Click here for file

Additional file 3 **Chronogram showing the posterior divergence ages within Murinae**. The topology corresponds with the ML tree in Figure 1. Divergence times have been estimated from the concatenated Cytochrome b, IRBP and GHR sequences by a Bayesian relaxed molecular clock method with two fossil calibration time constraints (nodes indicated by a star). Click here for file

Additional file 4 Estimated dates of divergence (Mya), standard deviation (SD) and 95% credibility intervals (CD) for selected nodes in Figure 2 and additional file 3 based on Bayesian approximation from the concatenation of the three genes and for each gene separately. Click here for file
