# Supplementary figures and images for: Phylogeny and biogeography of African Murinae based on mitochondrial and nuclear gene sequences, with a new tribal classification of the subfamily
Source: BMC Evol Biol. 2008 Jul 10;8:199. doi: 10.1186/1471-2148-8-199 (PMC2490707; doi:10.1186/1471-2148-8-199)

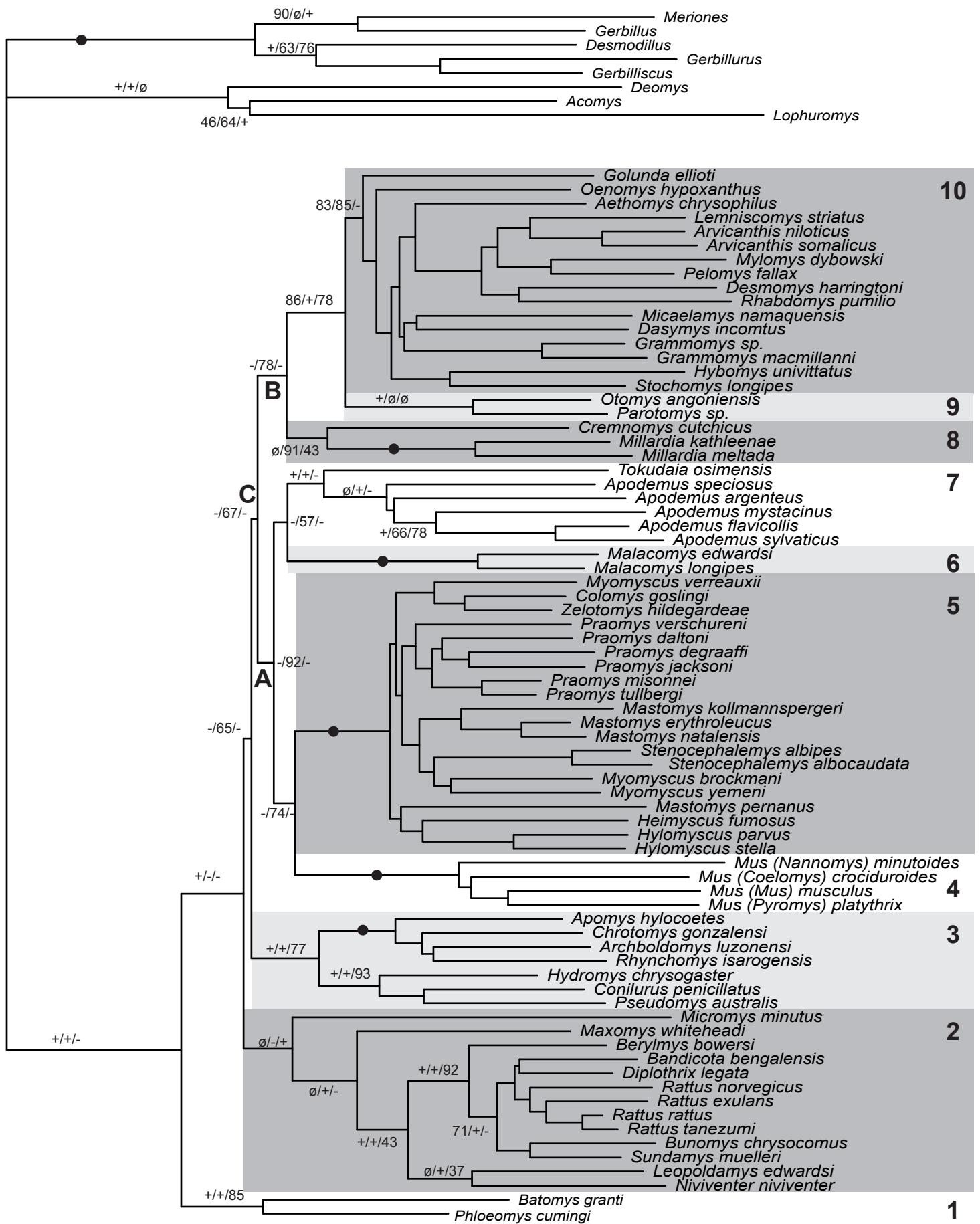

Supplement: Additional file 1 — Maximum likelihood topology obtained with the combined dataset. The support values from each gene separately are indicated for the main nodes discussed in the text. The support values are indicated as follow: GHR/IRBP/cytb. A black dot indicate that the node is supported by the three dataset with a BP > 95, +: BP > 95 otherwise the BP value is indicated, ø: no data available, -: not supported by the dataset. [file 1471-2148-8-199-S1.pdf]

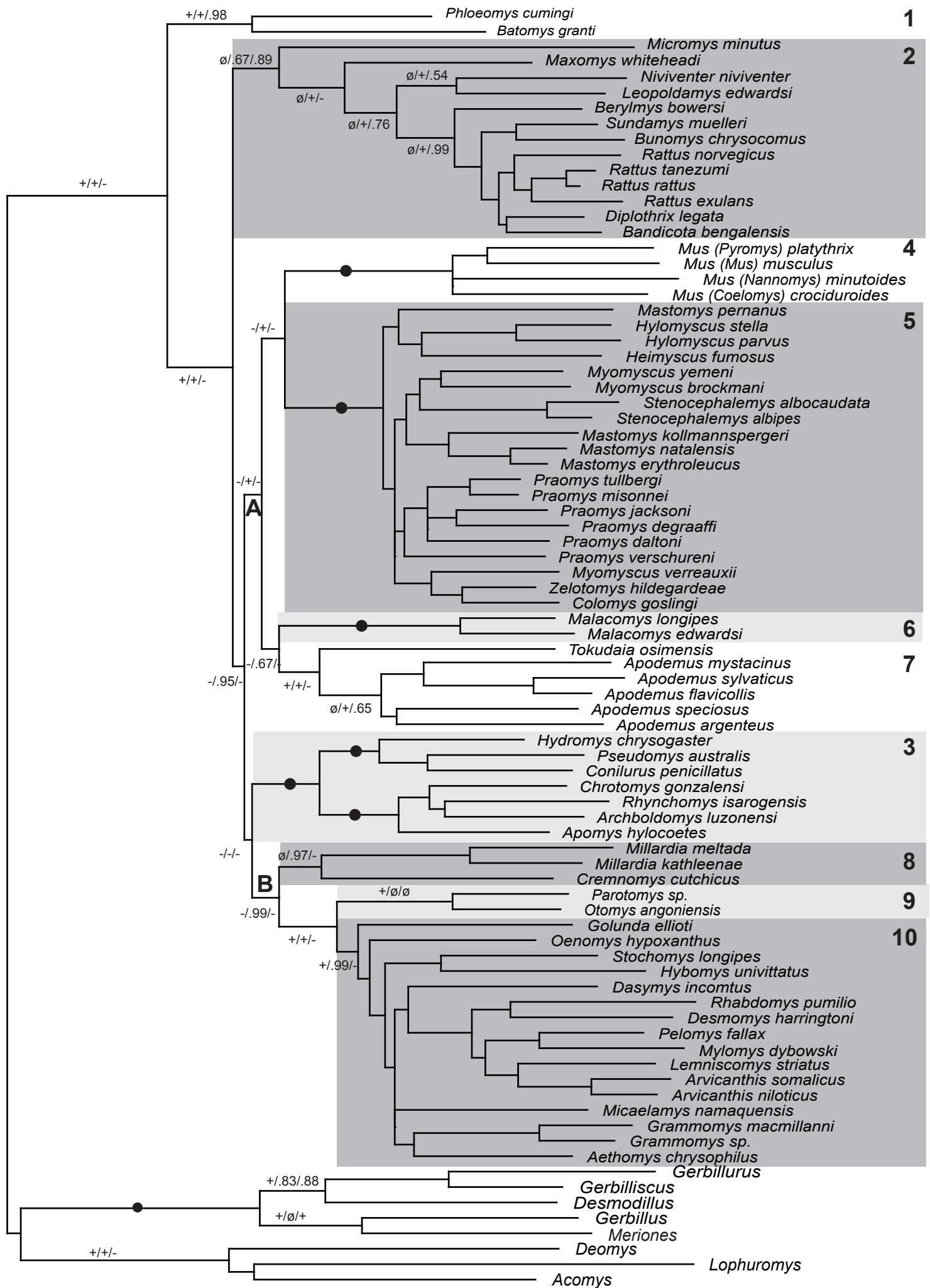

Supplement: Additional file 2 — Bayesian topology obtained with the combined dataset. The support values from each gene separately are indicated for the main nodes discussed in the text. The support values are indicated as follow: GHR/IRBP/cytb. A black dot indicate that the node is supported by the three dataset with a BP > 95, +: BP > 95 otherwise the BP value is indicated, ø: no data available, -: not supported by the dataset. [file 1471-2148-8-199-S2.pdf]

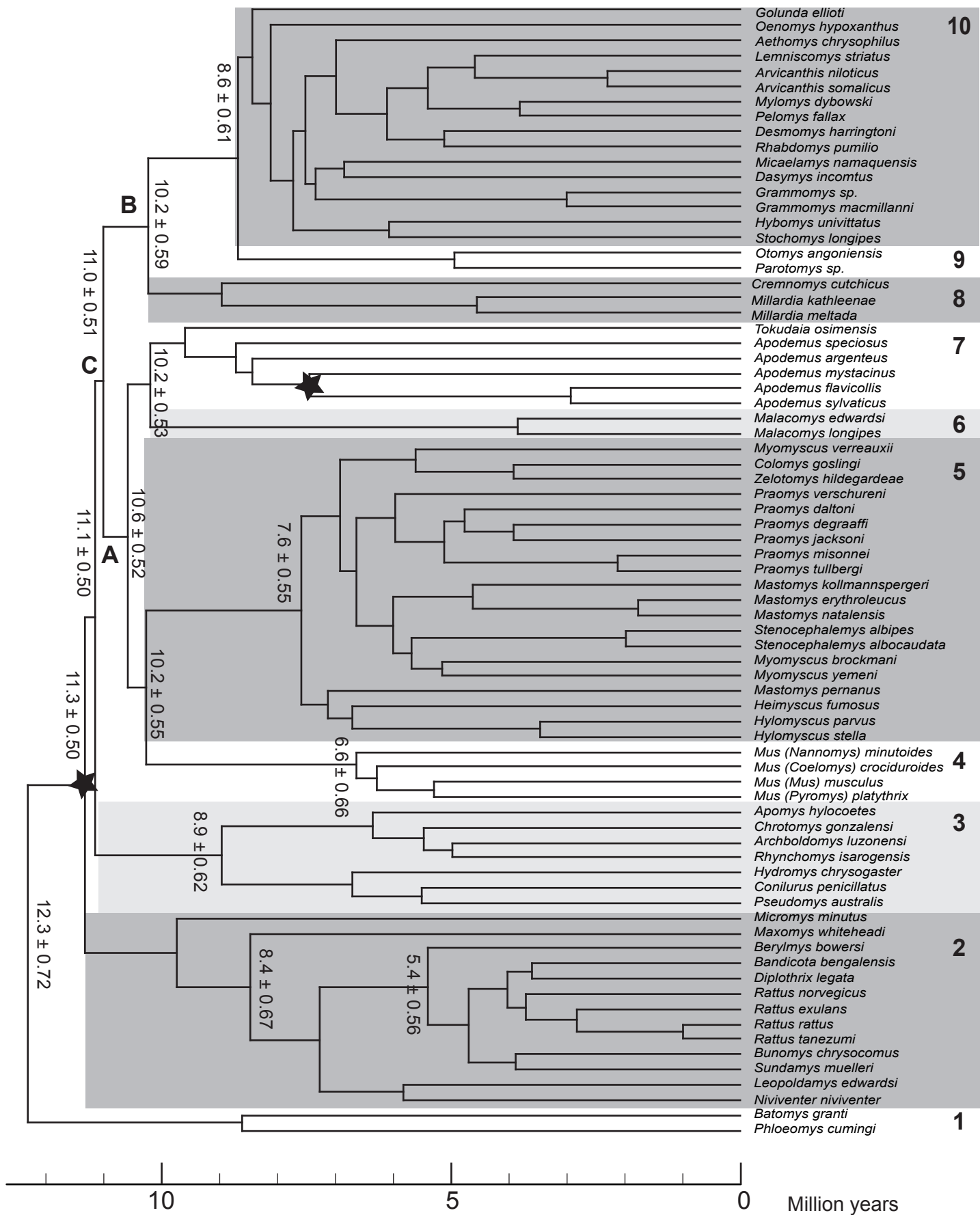

Supplement: Additional file 3 — Chronogram showing the posterior divergence ages within Murinae. The topology corresponds with the ML tree in Figure 1. Divergence times have been estimated from the concatenated Cytochrome b, IRBP and GHR sequences by a Bayesian relaxed molecular clock method with two fossil calibration time constraints (nodes indicated by a star). [file 1471-2148-8-199-S3.pdf]
