# Supplementary material for: Phylogeny and biogeography of African Murinae based on mitochondrial and nuclear gene sequences, with a new tribal classification of the subfamily
Source: BMC Evol Biol. 2008 Jul 10;8:199. doi: 10.1186/1471-2148-8-199 (PMC2490707; doi:10.1186/1471-2148-8-199)
Supplement: Additional file 4 — Estimated dates of divergence (Mya), standard deviation (SD) and 95% credibility intervals (CD) for selected nodes in Figure 2 and additional file 3 based on Bayesian approximation from the concatenation of the three genes and for each gene separately. [file 1471-2148-8-199-S4.doc]

|  | Combined dataset | | Cytb | | IRBP | | GHR | |
| --- | --- | --- | --- | --- | --- | --- | --- | --- |
| Node | Age ± SD | CD | Age ± SD | CD | Age ± SD | CD | Age ± SD | CD |
| Murinae | 12.3 ± 0.72 | 10.9-13.7 | 12.1 ± 0.68 | 10.8-13.5 | 12.3 ± 0.83 | 10.8-14.1 | 13.1 ± 1.4 | 11-16.5 |
| *Phloeomys/Batomys* | 8.6 ± 0.87 | 6.9-10.4 | 8.6 ± 1.1 | 6.6-10.9 | 8. 8 ± 1.3 | 6.4-11.5 | 7.8 ± 1.72 | 4.8-11.4 |
| Rattini/other Murinae | 11.3 ± 0.50 | 10.2-12 | 11.4 ± 0.46 | 10.3-12 | 11.4 ± 0.45 | 10.3-12 | 11.4 ± 0.48 | 10.2-12 |
| *Micromys*/other Rattini | 9.7 ± 0.69 | 8.4-11 | 9.2 ± 0.76 | 7.7-10.7 | 10.9 ± 0.62 | 9.5-11.8 | NA | NA |
| *Rattus* sensu lato | 8.4 ± 0.67 | 7.1-9.7 | 8.4 ± 0.72 | 7.1-9.9 | 8.5 ± 1.17 | 6.2-10.7 | NA | NA |
| *Rattus* sensu stricto | 5.4 ± 0.56 | 4.3-6.5 | 5.7 ± 0.65 | 4.5-7.1 | 4.9 ± 1.1 | 3-7.2 | 4.5 ± 1.11 | 2.6-7 |
| Hydromyini/other Murinae | 11.1 ± 0.50 | 10.0-11.9 | 11.2 ± 0.47 | 10.1-11.9 | 11.1 ± 0.51 | 10-11.9 | 11.1 ± 0.54 | 9.9-11.9 |
| Intra-Hydromyini (Philippines/Australia) | 8.9 ± 0.62 | 7.7-10.1 | 9.3 ± 0.72 | 7.9-10.7 | 8.89 ± 0.99 | 7-10.8 | 7.2 ± 1.19 | 4.9-9.6 |
| Clade C | 11.0 ± 0.51 | 9.9-11.8 | 11.1 ± 0.5 | 9.9-11.8 | 10.7 ± 0.56 | 9.5-11.7 | 10.7 ± 0.6 | 9.4-11.7 |
| Clade B | 10.2 ± 0.59 | 9.0-11.3 | 10.2 ± 0.62 | 8.9-11.3 | 9.8 ± 0.78 | 8.2-11.2 | 10.1 ± 0.72 | 8.6-11.3 |
| Otomyini/Arvicanthini | 8.6 ± 0.61 | 7.5-9.8 | 9.1 ± 0.67 | 7.7-10.4 | 8.3 ± 1.03 | 6.3-10.3 | 8.7 ± 0.9 | 6.9-10.4 |
| *Golunda*/African Arvicanthini | 8.4 ± 0.61 | 7.2-9.6 | 8.9 ± 0.67 | 7.6-10.2 | 7.2 ± 1.01 | 5.3-9.2 | 7.8 ± 0.94 | 6-9.7 |
| African Arvicanthini | 8.1 ± 0.60 | 6.9-9.2 | 8.5 ± 0.66 | 7.2-9.7 | 6.7 ± 0.99 | 4.8-8.7 | 7.4 ± 0.94 | 5.5-9.2 |
| Clade A | 10.6 ± 0.52 | 9.5-11.4 | 10.6 ± 0.52 | 9.5-11.5 | 10.2 ± 0.63 | 8.9-11.3 | 10.4 ± 0.6 | 9.1-11.5 |
| Malacomyini/Apodemyini | 10.2 ± 0.53 | 9.1-11.1 | 10.3 ± 0.54 | 9.1-11.2 | 9.8 ± 0.66 | 8.5-11 | 9.7 ± 0.75 | 8.1-11 |
| *Tokudaia*/*Apodemus* | 9.6 ± 0.54 | 8.5-10.6 | 9.8 ± 0.58 | 8.6-10. 9 | 9.1 ± 0.68 | 7.9-10.5 | 8.4 ± 0.76 | 7.2-10 |
| Praomyini/Murini | 10.2 ± 0.55 | 9.1-11.2 | 10.2 ± 0.55 | 9.1-11.2 | 9.3 ± 0.84 | 7.6-10.8 | 9.5 ± 0.85 | 7.7-11 |
| Praomyini | 7.6 ± 0.55 | 6.5-8.7 | 8.4 ± 0.64 | 7.1-9.7 | 6 ± 0.99 | 4.2-8.1 | 6.2 ± 1.09 | 4.2-8.43 |
| Murini | 6.6 ± 0.66 | 5.3-7.9 | 7.0 ± 0.78 | 5.6-8.6 | 5.7 ± 1.36 | 3.3-8.5 | 6.3 ± 1.4 | 3.7-9.1 |
